# PEDF Improves Cardiac Function in Rats with Acute Myocardial Infarction via Inhibiting Vascular Permeability and Cardiomyocyte Apoptosis

**DOI:** 10.3390/ijms16035618

**Published:** 2015-03-11

**Authors:** Hao Zhang, Zheng Wang, Shou-Jie Feng, Lei Xu, He-Xian Shi, Li-Li Chen, Guang-Da Yuan, Wei Yan, Wei Zhuang, Yi-Qian Zhang, Zhong-Ming Zhang, Hong-Yan Dong

**Affiliations:** 1 Department of Thoracic Cardiovascular Surgery, Affiliated Hospital of Xuzhou Medical College, Xuzhou 221006, China; E-Mails: zhanghao@xzmc.edu.cn (H.Z.); hefengx@163.com (Z.W.); 15152117880@163.com (S.-J.F.); 18796248806@163.com (L.X.); sunteng00@126.com (H.-X.S.); jxys139615@sina.com (L.-L.C.); yuanguangda5157@163.com (G.-D.Y.); xiaofengshen1109@163.com (W.Y.); xuyizyq@163.com (Y.-Q.Z.); 2 Research Facility Center for Morphology, Xuzhou Medical College, Xuzhou 221004, China; E-Mail: redwolf07@163.com

**Keywords:** pigment epithelium-derived factor (PEDF), myocardial infarction, cardiac function, vascular permeability, PPARγ, apoptosis

## Abstract

Pigment epithelium-derived factor (PEDF) is a pleiotropic gene with anti-inflammatory, antioxidant and anti-angiogenic properties. However, recent reports about the effects of PEDF on cardiomyocytes are controversial, and it is not known whether and how PEDF acts to inhibit hypoxic or ischemic endothelial injury in the heart. In the present study, adult Sprague-Dawley rat models of acute myocardial infarction (AMI) were surgically established. PEDF-small interfering RNA (siRNA)-lentivirus (PEDF-RNAi-LV) or PEDF-LV was delivered into the myocardium along the infarct border to knockdown or overexpress PEDF, respectively. Vascular permeability, cardiomyocyte apoptosis, myocardial infarct size and animal cardiac function were analyzed. We also evaluated PEDF’s effect on the suppression of the endothelial permeability and cardiomyocyte apoptosis under hypoxia *in vitro*. The results indicated that PEDF significantly suppressed the vascular permeability and inhibited hypoxia-induced endothelial permeability through PPARγ-dependent tight junction (TJ) production. PEDF protected cardiomyocytes against ischemia or hypoxia-induced cell apoptosis both *in vivo* and *in vitro* via preventing the activation of caspase-3. We also found that PEDF significantly reduced myocardial infarct size and enhanced cardiac function in rats with AMI. These data suggest that PEDF could protect cardiac function from ischemic injury, at least by means of reducing vascular permeability, cardiomyocyte apoptosis and myocardial infarct size.

## 1. Introduction

Myocardial infarction (MI) remains a major cause of morbidity and mortality despite that marked improvements in medical and device therapy have been achieved [1]. MI causes the dysfunction of cardiomyocytes, enhancement of vascular permeability, myocardial fibrosis and left ventricular remodeling. Indeed, cardiomyocyte apoptosis and vascular permeability are involved in myocardial fibrosis and LV remodeling, thus promoting contractile dysfunction [2]. After these effects, irreversible damage occurs in the ischemic heart.

Pigment epithelium-derived factor (PEDF), a non-inhibitory member of the serine protease inhibitor superfamily (SERPINS), is a glycoprotein with a molecular weight of 50 kDa identified in various human tissues, such as eye, brain, muscle, adipose tissue, liver and bone [3,4,5]. PEDF is characterized as a multifunctional protein possessing antiangiogenic, antitumorigenic, antioxidant, anti-inflammatory, antithrombotic, neurotrophic and neuroprotective properties [6,7,8,9]. PEDF regulates vascular permeability by preventing the dissociation of endothelial adherens junctions (AJs) and tight junctions (TJs) via a γ-secretase-dependent mechanism in retinal microvascular endothelial cells [10]. Recently, PEDF mRNA and protein have been demonstrated to be expressed in the human heart and secreted by cardiomyocytes and fibroblasts [11]. PEDF may rely on any of its antioxidant, anti-inflammatory and antithrombotic protective properties to manifest a counteractive mechanism during the development of atherosclerosis and cardiovascular disease [12].

The findings mentioned above led us to speculate that PEDF could exert beneficial effects on cardiac function by suppressing vascular permeability and cardiomyocyte apoptosis in acute myocardial infarction (AMI). However, the protective role of PEDF against vascular permeability after AMI remains unknown, and some investigations about the effect of PEDF on cardiomyocytes were controversial. Ueda, S. *et al*. [13] demonstrated that PEDF inhibits tissue remodeling and improves cardiac function in the rat AMI model by reducing the death of apoptotic cells, oxidative stress generation and suppressing cardiac fibrosis. Nevertheless, Li *et al*. [14] found that PEDF increased cardiomyocyte apoptosis during hypoxia via the Fas signaling pathway. Kathrin Rychli’s study revealed that PEDF may contribute to the progression of heart failure by inducing apoptosis in human cardiomyocytes and fibroblasts via activation of caspase-3 [15].

Therefore, in this study, we delivered lentivirus carrying PEDF or PEDF RNAi by using intramyocardial injections to overexpress or knockdown PEDF in a rat AMI model. We aimed to investigate whether PEDF could inhibit vascular permeability and cardiomyocyte apoptosis and improve cardiac function in rats with AMI and tried to elucidate the possible mechanisms.

## 2. Results

### 2.1. PEDF Improves Cardiac Function

**Figure 1:**
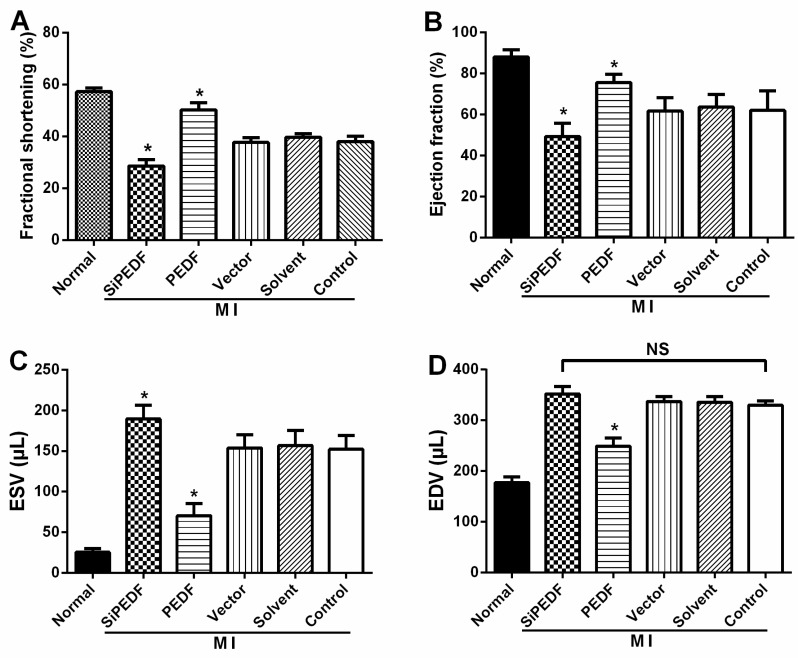

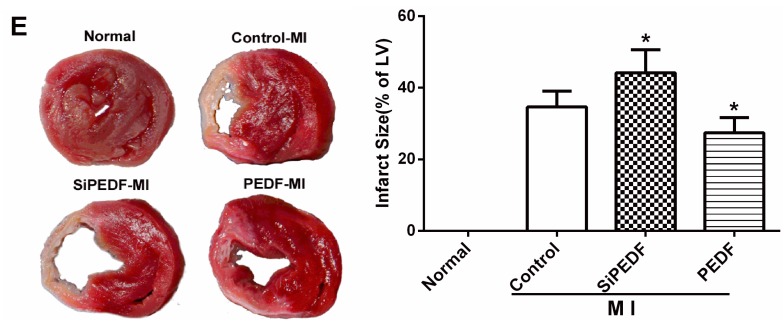
Myocardial infarct size of the left ventricle and cardiac function determination. (**A**) Left ventricular fractional shortening; (**B**) Left ventricular ejection fraction; (**C**) Left ventricular end-systolic volume (ESV); (**D**) Left ventricular end-diastolic volume (EDV); (**E**) Representative figures of the 2,3,5-triphenyltetrazolium (TTC)-stained myocardial tissues at four weeks after acute myocardial infarction (AMI) in each indicated experimental condition and quantification of the infarct size. Data are expressed as the mean ± SD (*n* = 6). * *p* < 0.05 *vs.* the control group; NS, No statistical difference; MI, myocardial infarction; PEDF: pigment epithelium-derived factor.

Cardiac function was examined using echocardiography four weeks after AMI. Significant improvement in cardiac systolic function in the PEDF group was present four weeks after AMI, and the values of Left ventricular fractional shortening (FS)% and Left ventricular ejection fraction (EF)% in this group were significantly larger than those of the siPEDF and the other control groups (*p* < 0.05) (Figure 1A,B). Moreover, the values of Left ventricular end-systolic volume (ESV) and Left ventricular end-diastolic volume (EDV) in the PEDF group were also significantly decreased compared to the siPEDF and other control groups (*p* < 0.05). However, we did not find any significant difference in EDV between the siPEDF and ischemia control groups (*p* > 0.05) (Figure 1C,D). These results clearly indicated that the cardiac systolic function in the PEDF group was remarkably improved.

### 2.2. PEDF Reduces Myocardial Infarct Size

PEDF treatment significantly decreased myocardial infarct size (MIS) compared to the vector control four weeks after AMI. However, the MIS percentage was markedly increased in the siPEDF-treated groups compared to in the vector control rats (Figure 1E). These results suggest that PEDF reduces the infarct size during the development of ischemic injury in an AMI rat model.

### 2.3. PEDF Reduces Cardiomyocyte Apoptosis in the Ischemic Myocardium

**Figure 2:**
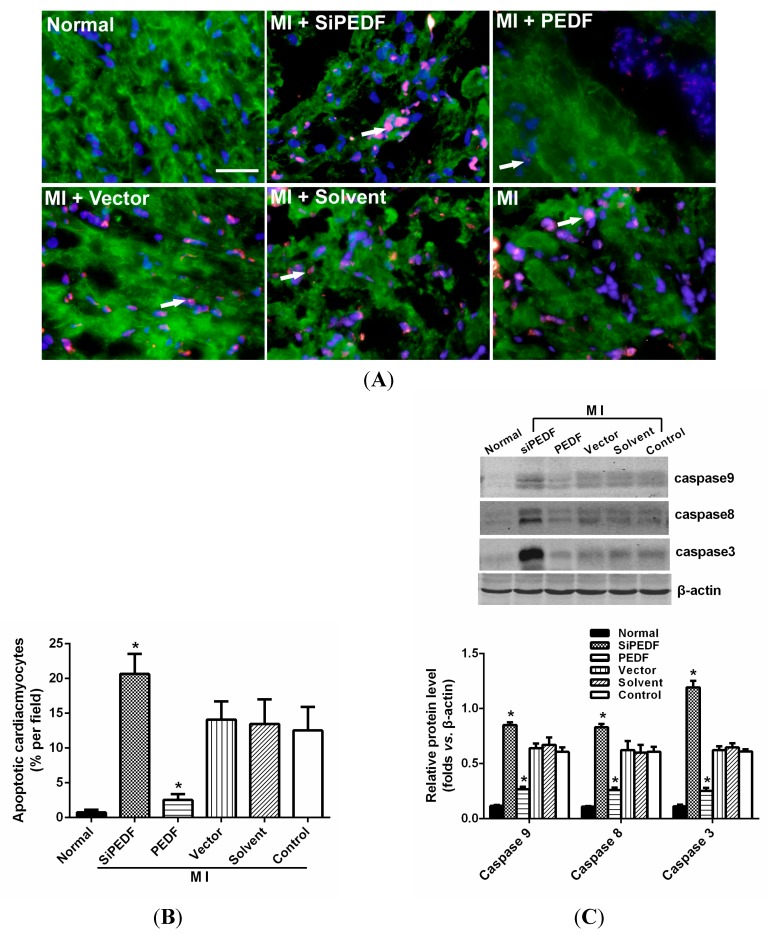
Cardiomyocyte apoptosis assay *in vivo*. (**A**) Representative images of TUNEL staining of cardiomyocyte apoptosis (white arrow). Cardiomyocyte apoptosis was measured by TUNEL staining; cardiomyocytes were identified using α-sarcomeric actinin antibodies (α-sa). TUNEL staining for cardiomyocyte apoptosis (red), Hoechst for nuclear staining (blue) and α-sa for cardiomyocytes (green) in the border zone of the infarcted left ventricle (LV) at four weeks after AMI (Scale bar = 20 μm); (**B**) Bar graphs indicate quantitative analysis of cardiomyocyte apoptosis; and (**C**) Western blot determination for protein expression of cleaved caspases-8, -9, and -3 and FasL. Data are expressed as the mean ± SD (*n* = 6). * *p* < 0.05 *vs.* the control group.

The results of terminal deoxynucleotidyl transferase-mediated dUTP nick end labeling (TUNEL) staining showed that the amount of cardiomyocyte apoptosis was significantly increased in the siPEDF group four weeks after AMI (*p* < 0.05), whereas the amount of cardiomyocyte apoptosis in the PEDF group was reduced when compared with the other groups (*p* < 0.05) (Figure 2A,B). In addition, we further determined the expression of proteins related to apoptotic signaling pathways. As shown in Figure 2C, in the siPEDF group, increased expression of cleaved caspases-8, -9, and -3 and FasL protein was evident (*p* < 0.05). However, in the PEDF group, cleaved caspases-8, -9, and -3 and FasL protein expression was significantly decreased when compared with the control groups (*p* < 0.05).

### 2.4. PEDF Protects against Hypoxia-Induced Cardiomyocyte Apoptosis

**Figure 3:**
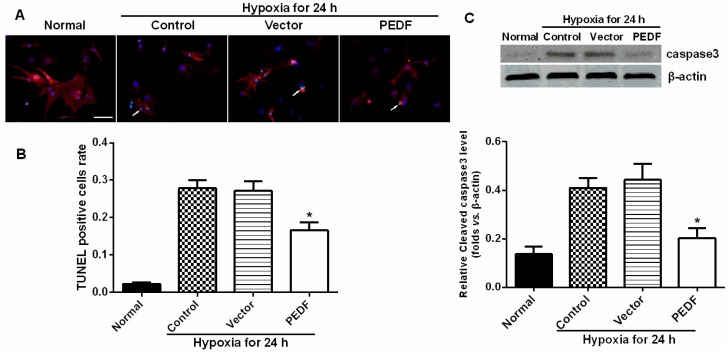
PEDF protected against hypoxia-induced cell death. (**A**) TUNEL (green) and α-sa (red) staining were performed for each group. Nuclei were stained with Hoechst (blue). Immunofluorescent images showed that TUNEL-positive cells (indicated by the arrows) were increased after hypoxia. Under condition of hypoxia, PEDF exhibited anti-apoptotic effects (scale bar = 20 μm); (**B**) Statistical analyses from immunofluorescent images; (**C**) Western blot determination for protein expression of cleaved caspases-3. The cleaved caspase-3 protein level was also decreased in the PEDF groups (hypoxia for 24 h). Data are expressed as the mean ± SD (*n* = 6). * *p* < 0.05 *vs.* the control group.

We further verified the protective effects of PEDF on apoptosis in cultured neonatal rat myocardial cells. Under condition of hypoxia, PEDF exhibited anti-apoptotic effects (*p* < 0.05), and the cleaved caspase-3 protein level was also decreased in the PEDF groups (hypoxia for 24 h) (Figure 3). These results suggest that PEDF inhibits the expression of cleaved caspase-3, and PEDF protects neonatal rat myocardial cells against hypoxia-induced cell apoptosis.

### 2.5. PEDF Prevents Vascular Leakage

**Figure 4:**
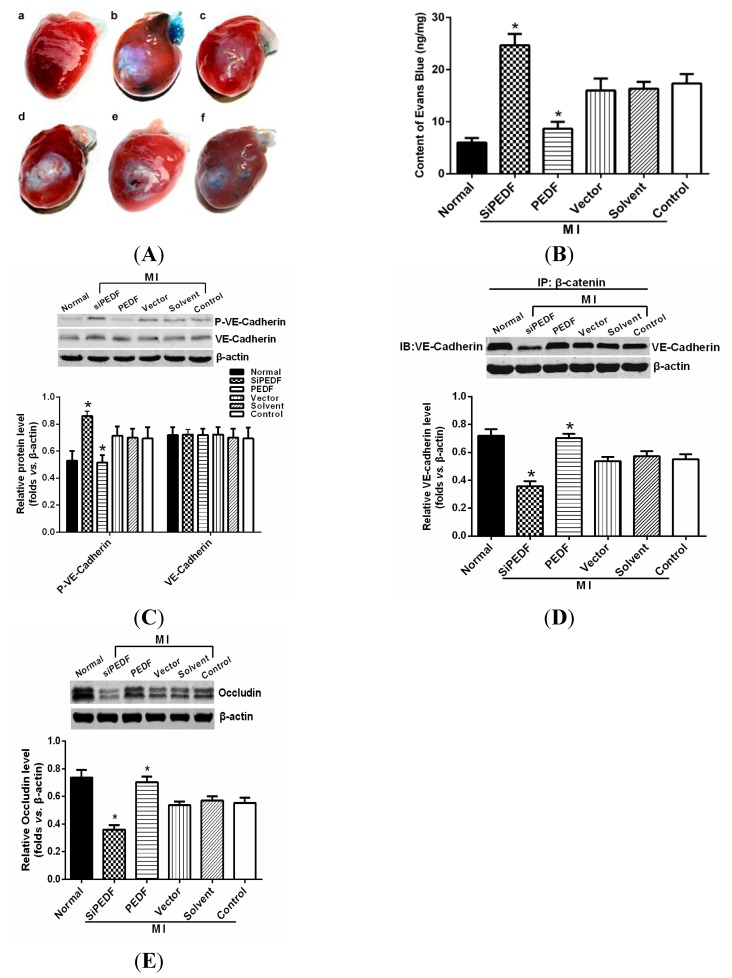
Vascular permeability in the ischemic heart. (**A**) The representative figures show the following: (a) normal, (b) SiPEDF, (c) PEDF, (d) vector control, (e) solvent control and (f) ischemic control; (**B**) Quantification of the Evans Blue content; (**C**) Western blot determination of VE-cadherin/VE-cadherin tyrosine phosphorylation; (**D**) Co-immunoprecipitation of VE-cadherin/β-catenin complexe; and (**E**) Western blot determination of occludin. Data are expressed as the mean ± SD (*n* = 6). * *p* < 0.05 *vs.* the control group.

The normal heart was dye-free. The amount of dye that leaked out of the coronary artery and infiltrated the myocardium in the PEDF group was significantly less than the amounts in the siPEDF, vector, solvent and ischemic control groups (*p* < 0.05). However, a large quantity of Evans Blue residing in the myocardium was detected in the siPEDF group (Figure 4A,B).

To further determine whether vascular permeability was affected by PEDF expression, we examined the changes in the main functional proteins of AJs (VE-cadherin and β-catenin) and TJs (occluding). No significant difference was detected among the groups with regard to VE-cadherin expression. The level of VE-cadherin tyrosine protein phosphorylation was high in the siPEDF group and was low in the PEDF group (Figure 4C). The results of co-immunoprecipitation (co-IP) showed that the level of VE-cadherin associated with β-catenin was significantly low in the siPEDF group (*p* < 0.05). In contrast, a high level of VE-cadherin expression was found in the PEDF group (Figure 4D). We also found that occludin protein expression was decreased in the siPEDF group and was increased in the PEDF group (Figure 4E).

### 2.6. PEDF Prevents Hypoxia-Induced Rat Aortic Endothelial Cell Permeability

**Figure 5:**
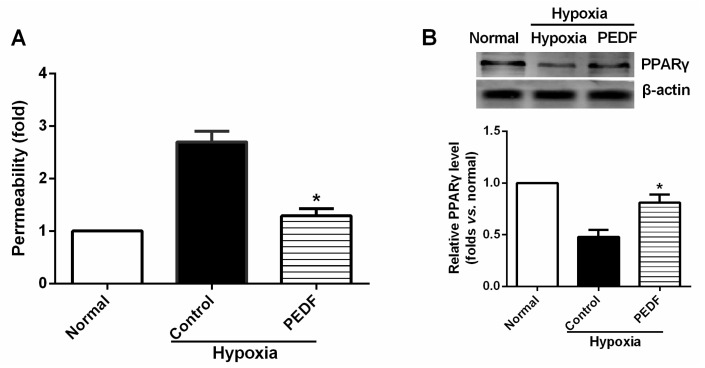
PEDF prevents permeability and increases the expression of PPARγ under hypoxia in rat aortic endothelial cells (RAECs). Endothelial permeability (**A**) was determined. PEDF significantly reduced hypoxia-induced RAECs permeability (*n* = 6); and (**B**) PEDF increased the expression of PPARγ under hypoxia in RAECs (*n* = 3). Data shown in (**A**) and (**B**) are presented as mean ± SD. * *p* < 0.05 *vs.* the control group.

We began by examining whether PEDF would inhibit endothelial permeability under hypoxia in rat aortic endothelial cells (RAECs). For this, RAECs were exposed to normoxia or hypoxia for 24 h in the absence or presence of PEDF and measured for permeability. Hypoxia-induced increases of endothelial permeability were abolished with PEDF treatment (Figure 5A), suggesting that PEDF significantly reduced hypoxia-induced RAECs permeability.

It has been reported that peroxisome proliferator activated γ (PPARγ) plays a critical role in the control of the barrier functions of epithelial and endothelial cells [16,17], and PEDF can also increase the expression and transcriptional activity of PPARγ in THP-1 macrophages [18]. To investigate whether PPARγ was a major regulator of the signaling pathway by which PEDF inhibits endothelial permeability, we examined the PPARγ expression after PEDF treatment under hypoxia. As shown in Figure 5B, PPARγ protein expression in the hypoxia group was significantly inhibited, whereas the expression level of PPARγ protein was increased notably in the PEDF group (*p* < 0.05). Our results indicate that PEDF can also increase the expression of PPARγ in RAECs.

### 2.7. PEDF Inhibits Hypoxia-Induced RAECs Permeability through PPARγ-Dependent TJ Production

**Figure 6:**
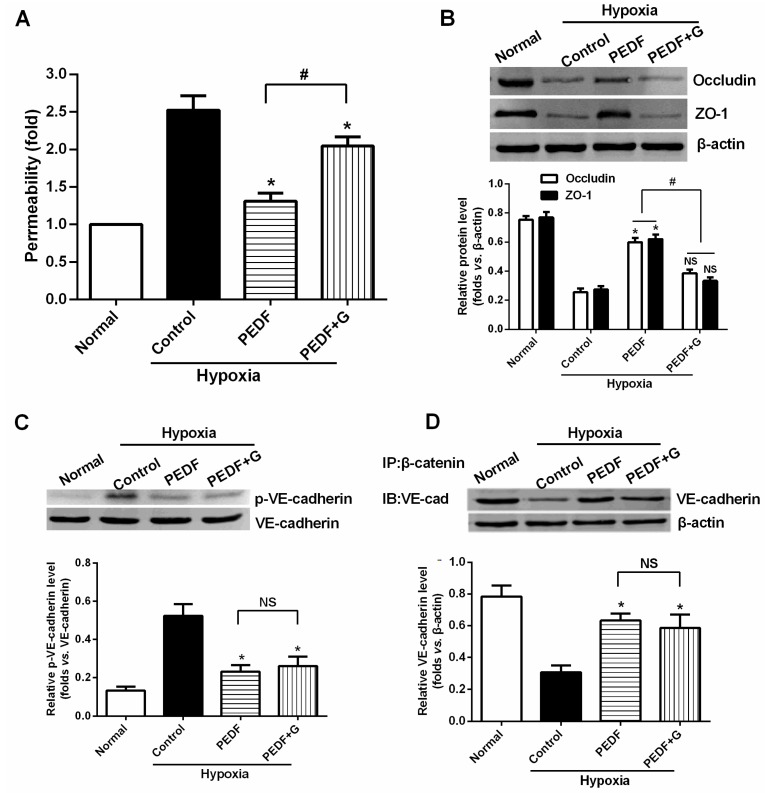
PEDF inhibits hypoxia-induced RAEC permeability through PPARγ-dependent tight junction (TJ) production. (**A**) Endothelial permeability determination. PEDF’s effect on the suppression of the endothelial permeability was significantly lost in the presence of PPARγ inhibitor GW9662. Data are expressed as the mean ± SD (*n* = 6). * *p* < 0.05 *vs.* the control group, ^#^ *p* > 0.05; (**B**) Western blot determination of TJs (occludin and ZO-1). * *p* < 0.05 *vs.* the control group, ^#^ *p* < 0.05; NS, *p* > 0.05 *vs.* the control group (*n* = 3); (**C**) Western blot determination of VE-cadherin/VE-cadherin tyrosine phosphorylation. * *p* < 0.05 *vs.* the control group; NS, *p* > 0.05 (*n* = 3); and (**D**) Co-immunoprecipitation of VE-cadherin/β-catenin complexes. * *p* < 0.05 *vs.* the control group; NS, No statistical difference (*n* = 3). These findings suggest that PEDF inhibits RAEC permeability under hypoxia via enhancing the expression of TJs and adherens junctions (AJs). PEDF inhibits hypoxia-induced RAECs permeability through PPARγ-dependent TJ production (*n* = 3).

Likewise, when we added PPARγ inhibitor GW9662, PEDF’s effect on the suppression of the endothelial permeability was significantly lost (Figure 6A). These findings suggest that PEDF inhibits RAEC permeability under hypoxia through PPARγ. This hypothesis was further tested by additional experiments using Western blot. As shown in the figure, Western blots of RAECs lysates under hypoxia treated with PEDF showed significant upregulation of TJs (occludin and ZO-1) compared with the hypoxia group. However, the upregulation of occludin and ZO-1 proteins retracted in the presence of PPARγ inhibitor GW9662 (Figure 6B). The level of VE-cadherin tyrosine phosphorylation was high in the hypoxia group, but was low in the PEDF group (Figure 6C). The results of co-IP showed that the level of VE-cadherin associated with β-catenin was low in the hypoxia group, but was significantly high in the PEDF group (Figure 6D). Under these conditions, when we added PPARγ inhibitor GW9662, PEDF’s effects on the AJs (VE-cadherin and β-catenin) were nearly unchanged (Figure 6C,D). These findings suggest that PEDF inhibits RAECs permeability under hypoxia via enhancing the expression of TJs and AJs. However, TJs may represent a major role by which PEDF inhibits RAEC permeability through PPARγ.

## 3. Discussion

Recent investigations have suggested that PEDF treatment can potently inhibit tissue remodeling and improve cardiac function in the rat AMI model [13] and suppress retinal microvascular permeability [10]. However, to date, it is not known whether and how PEDF acts to inhibit hypoxic or ischemic endothelial injury in the heart. Meanwhile, some investigations about the effect of PEDF on cardiomyocytes were controversial. In this study, we reported that although PEDF inhibited angiogenesis in the infarct border zone, PEDF reduced vascular permeability and infarct size, protected cardiomyocytes in an ischemic heart and improved functional recovery in an AMI rat model. We also discovered a new and beneficial role for PEDF, which prevented hypoxia and ischemia-induced endothelial barrier dysfunction and revealed the molecular basis for PEDF-mediated protection of hypoxic RAECs. To the best of our knowledge, this is the first report delineating the role of PEDF in the regulation of vascular permeability in RAECs via PPARγ under the hypoxic condition. In addition, we found that PEDF protected cardiomyocytes against ischemia or hypoxia-induced cell apoptosis, both *in vivo* and *in vitro*, via preventing the activation of caspase-3.

We observed that PEDF was downregulated in the peri-infarct region after AMI. Thus, to verify the role of PEDF in ischemic heart, we delivered lentivirus carrying PEDF or PEDF RNAi by using intramyocardial injections to overexpress or knockdown PEDF in a rat AMI model (Supplementary Figure S1). In ischemic heart, the vascular permeability was increased with vascular perfusion dysfunction, which was not conducive to functional recovery of AMI. We found that silencing of PEDF significantly decreased the binding of VE-cadherin with β-catenin and the expression of occludin, resulting in destabilized endothelial junctions and high vascular permeability in an AMI rat model. Our results are consistent with the studies that PEDF inhibits vascular permeability in retinal micro-vessels [10]. Thus, although PEDF had a negative effect on angiogenesis, it effectively reduced local vascular permeability, indicating that PEDF may improve functional recovery in the ischemic heart.

The most important finding of this study is that PEDF-mediated PPARγ production in hypoxic RAECs promotes the upregulation of TJs, leading to a sustained barrier function. Such observations may be important to ensure that PPARγ is an important regulator of the signaling pathway by which PEDF inhibits endothelial permeability. Although the exact mechanism remains elusive, some recent studies have suggested that activation of PPARγ stimulates lipid synthesis, epidermal differentiation and aquaporin 3 expression and accelerates recovery from permeability barrier dysfunction [19]. PPARγ agonists upregulate the barrier function of tight junctions via a PKC pathway in human nasal epithelial cells [17]. Furthermore, when we added PPARγ inhibitor GW9662, PEDF was unable to suppress hypoxia-promoted permeability effectively in RAECs, and PEDF-mediated upregulation of occludin and ZO-1 proteins retracted. Interestingly, PEDF’s effects on the AJs were nearly unchanged in the presence of PPARγ inhibitor GW9662. These findings suggest that PEDF inhibits RAEC permeability under hypoxia via enhancing the expression of TJs and AJs. However, TJs may represent a major role by which PEDF inhibits RAEC permeability through PPARγ. The effects of PEDF upregulating AJs may be via the other path. Consequently, further study is needed to address the issue.

It is widely recognized that cardiomyocyte death is an important component in the pathogenesis of myocardial infarction and heart failure [20]. Pathologically, failing hearts after AMI exhibit ongoing cardiomyocyte death over months to years at levels that are low, but still 100-fold higher than those observed in normal hearts [21]. Our study showed that knocking down PEDF increased cardiomyocyte apoptosis, whereas PEDF overexpression decreased the apoptosis. The expression levels of FASL and caspase-8, crucial triggers in the death receptor domain signal pathway, were increased in the peri-infarct region after initiating AMI. Meanwhile, the expression of caspase-9 was also increased, which subsequently activated the mitochondrial apoptotic pathway. We found that the activation of these two apoptotic pathways was remarkably enhanced once PEDF expression was silenced. In contrast, PEDF overexpression effectively inhibited these two apoptotic pathways, thus presenting an anti-apoptotic property. Our results demonstrate that PEDF may enhance cardiomyocyte survival by protecting cardiomyocytes against apoptosis in the ischemic heart.

In addition, we also found that PEDF protected cardiomyocytes against hypoxia-induced cell apoptosis via preventing the activation of caspase-3. However, Li *et al*. [14] reported that PEDF (200 ng/mL) increased cardiomyocyte apoptosis under both hypoxic and normoxic conditions. This conclusion conflicts with Ueda’s finding [13]*,* and Ueda *et al*. demonstrated that a concentration of PEDF close to 200 ng/mL in normal serum caused no impairment of cardiomyocyte survival. Recently, several studies presented proofs of concept of yet another therapeutic role for PEDF, that of retinal neuroprotection from oxidative stress, and provided evidence of a potential effect on the broader area of apoptosis-mediated cell death [22]. Oxidative stress is also a mediator of cardiomyocyte apoptosis. Our previous studies also found that PEDF protected cultured H9c2 cells against apoptosis and necroptosis under hypoxic condition via the anti-oxidative mechanism [23]. The result matches that of the *in vitro* experiments in this study.

There may be some relationship between the events of vascular permeability and myocardial apoptosis. The effect of anti-permeability to inhibit cardiac inflammation and maintain the cardiac environment may represent an important mechanism that further links cardiomyocyte protection to cardiovascular disease. Furthermore, we evidenced that PEDF reduced the infarct size, resulting in functional recovery in an AMI rat model. Given the fact that PEDF levels were decreased in the peri-infarct zone, our present study provides a novel therapeutic potential of the substitution of PEDF for cardiac dysfunction in AMI.

## 4. Experimental Section

### 4.1. Recombinant Lentivirus Constructs and Viral Production

Recombinant lentivirus (LV) was prepared as described previously. PEDF over-expression plasmids and the RNAi vector PEDF-RNAi-LV of the PEDF gene producing PEDF shRNA were successfully constructed and were then successfully packaged in 293T cells. The concentrated titer of virus suspension was 2 × 10^12^ TU/L.

### 4.2. Animal Model and Intramyocardial Gene Delivery

Sprague-Dawley male rats (8 to 10 weeks old, weighing 210–250 g, 230 ± 20 g) were obtained from the Experimental Animal Center of Xuzhou Medical College, Xuzhou, China. All of the experiments conform to the Guide for the Care and Use of Laboratory Animals published by the U.S. National Institutes of Health (NIH Publication, 8th Edition, 2011). The animal care and experimental protocols were approved by the Xuzhou Medical College Committee on Animal Care.

Myocardial ischemia was induced by ligation of the left-anterior descending coronary artery (LAD) in anesthetized rats, as described previously [24] (see the Supplementary Material, Methods 1.1). For intramyocardial gene delivery, PEDF-LV or PEDF-RNAi-LV (2 × 10^7^ TU) in 20 μL enhanced infection solution (ENIS, pH 7.4) was delivered with a 20-μL syringe and 25-gauge needle into the myocardium along the infarct border immediately after surgery. Control animals received an equivalent volume of either ENIS or lentivirus vector. The reliability of the injection transfection method was confirmed by the blue-stained myocardial size infiltrated by injected methylene blue (Supplementary Figure S2). The animal models were randomly divided into six groups: Group A (normal); Group B (siPEDF), PEDF-RNAi-LV was transferred after surgery; Group C (PEDF), PEDF-LV was transferred after surgery; Group D (vector control), LV vector was transferred into the ischemic myocardium; Group E (solvent control), 20 μL ENIS were transferred as a solvent control; and Group F (ischemic control), the animal did not undergo any gene transfer after surgery. The animals were sacrificed with an overdose of sodium pentobarbitone (60 mg/kg, i.v.), and their hearts were harvested at the end of 2 or 4 weeks after AMI for further analysis.

### 4.3. Animal Cardiac Function Evaluation

Echocardiography was conducted under sedation by sodium pentobarbital (30 mg/kg, i.p), as described previously [25]. Two-dimensional-guided (2D) M-mode echocardiography was used to determine left ventricular (LV) chamber volume at systole and diastole and contractile parameters, such as left ventricular end-diastolic dimension (LVEDD), left ventricular end-systolic dimension (LVESD), left ventricular end-diastolic volume (LVEDV) and left ventricular end-systolic volume (LVESV). The left ventricular fractional shortening (LVFS) was calculated as follows: FS (%) = (LVEDD − LVESD)/LVEDD × 100. The ejection fraction (EF) was then derived as EF (%) = (EDV – ESV)/EDV × 100.

### 4.4. Quantification of Myocardial Infarct Size

Four weeks after LAD artery ligation, the selected rats were euthanized, then the hearts were removed for MIS analyses by the method of 2,3,5-triphenyltetrazolium (TTC) staining [26]. The left ventricle (LV) was isolated and cut into 4-mm slices perpendicular to the axis of the LAD. Then, the slices were immediately immersed in 1% TTC (Sigma Chemical Co., St. Louis, MO, USA) in phosphate buffer (pH 7.4) at 37 °C for 10 min to discriminate the infarcted tissue from viable myocardium. All slices were scanned from both sides by a color CCD camera (FV-10, Fuji, Japan), and in each slide, the infarct area was compared with the total area by using digital planimetry software (Image-Pro Plus 6.0, Media Cybernetics Inc., Bethesda, MD, USA). After correction with the weight of the slices, the infarct size was calculated as a percentage of the LV.

### 4.5. Cardiomyocyte Apoptosis Assay in Vivo

The myocardial samples from the animal left ventricle were embedded in optimum cutting temperature (OCT) compound tissue medium (SAKURA, Tokyo, Japan), snap-frozen on dry ice and stored at −80 °C. Cardiomyocyte apoptosis *in vivo* was determined by double-labeling immunofluorescence staining of TUNEL, which was performed with an *in situ* cell death detection kit according to the manufacturer’s instructions using the In Situ Cell Death Detection Kit, POD (Cat. No, 11684817910, Roche Applied Science, Mannheim, Germany). Cardiomyocytes were identified using α-sarcomeric actinin antibodies (α-sa) (Sigma Chemicals). Hoechst (Sigma Aldrich) staining was used to count the total number of nuclei. Cardiomyocyte nuclei with a larger diameter can be recognized as being located within myofibers. Cardiomyocytes from at least three randomly selected sections or 48-well plates were evaluated for these variables. The number of TUNEL-positive cells was calculated as cells per area at 400-fold magnification. The percentage of TUNEL-positive cells was calculated as a percentage of total cells viewed in five randomly selected fields for each group.

### 4.6. Vascular Permeability Measurement

To determine vascular permeability in the rat hearts, a Miles assay was employed based on the method described previously [27,28]. Two percent Evans Blue dye (30 mg/kg, Sigma Chemicals) was injected intravenously 30 min before animal sacrifice. After sacrifice, the sample of heart tissue was weighed, and the Evans Blue dye was extracted by immersion in formamide (1 mL/100 mg) overnight at 60 °C. The extract was centrifuged at a speed of 7000 rpm for 45 min at 4 °C. The content of Evans Blue dye was determined on the basis of absorbance at 620 nm, and the ratio between the transferred and the intact normal heart samples was calculated and normalized to the tissue weight.

### 4.7. Western Blot Analysis and Co-Immunoprecipitation

Total proteins were extracted from the myocardium of the peri-infarct zone with ice-cold lysis buffer, as described previously [27]. Membranes were incubated with the following primary antibodies: PEDF, TNFα, IL-6 and FasL (Santa Cruz Biotechnology, Santa Cruz, CA, USA), ZO-1, p-VE-cadherin (Abcam, Cambridge, MA, USA); β-catenin, VE-cadherin, occludin, caspase-9, caspase-8 and caspase-3 (Anbo Biotechnology Co., Ltd., San Francisco, CA, USA). β-actin was used as a loading control. Co-immunoprecipitation (co-IP) was performed to examine the combination of VE-cadherin/β-catenin, as detailed in the Supplementary Materials (Methods 1.2).

### 4.8. Preparations of PEDF Protein

Recombinant rat PEDF (GenBankTM Accession Number NM_177927) was synthesized by Cusabio Biotech, Co., Ltd. (Wuhan, China). In brief, 293T cells were transfected with the recombinant vector pGEX 6P-1 GST-tagged PEDF. GST-tagged PEDF proteins were purified by high pressure liquid chromatography purification (>90% purity) and amino-terminal sequence determination. The resulting proteins were soluble in aqueous solutions.

### 4.9. Cardiomyocyte Culture and Cardiomyocyte Apoptosis Assay in Vitro

Neonatal Sprague-Dawley (SD) rats (1–3 days old, weighing 5–7 g, means 6.1 ± 0.7 g) were obtained from the Experimental Animal Centre of Xuzhou Medical College. All of the experiments conform to the guidelines as described before on the ethical use of animals. Ventricular myocytes were isolated from neonatal SD rats. The apoptosis assay was used to study four groups: a normal control group, a hypoxia group, a vector control (10 nmol/L) + hypoxia group and a PEDF-treated (10 nmol/L) + hypoxia group. The cardiomyocytes in the normal control group were maintained under normoxic conditions (95% air, 5% CO_2_) at 37 °C for 24 h. Cardiomyocytes in the hypoxia groups were exposed to hypoxic conditions (94% N_2_, 5% CO_2_, 1% O_2_) in an anaerobic system (Thermo Forma, Marietta, OH, USA) at 37 °C for equivalent periods. The cells were then harvested, and apoptotic cells were detected by TUNEL staining according to the manufacturer’s protocol.

### 4.10. RAECs Culture and Permeability Measurements in Vitro

RAECs were isolated and cultured as described previously [29]. Changes in the permeability of monolayer RAECs were examined using cell culture transwell inserts (12 mm diameter, 0.4 mm pore size; Transwell, Corning, Cambridge, MA, USA). Endothelial cells were maintained at confluence on porous polyester membrane inserts prior to experimentation. The upper and lower compartments contained 150 and 0.5 mL of media, respectively. PEDF (10 nmol/L) was added to both the upper and the lower chambers for 1 hour before the addition of 10 μM FITC-dextran to the upper chamber of the inserts. Then, RAECs were exposed to hypoxic conditions (94% N_2_, 5% CO_2_, 1% O_2_) in an anaerobic system (Thermo Forma) at 37 °C for 24 h, while the controls were left in normoxic conditions at 37 °C for the equivalent periods. A 50-μL sample of medium was taken in triplicate from the lower chamber and placed in 96-well cluster plates (black with clear bottoms, polystyrene; Corning Costar) to measure fluorescence intensity (excitation at 530 nm and emission at 590 nm). The FITC-dextran that passed across the endothelial cell monolayer was normalized to the fluorescence reading from the upper chamber, and permeability was calculated as relative fluorescence units.

### 4.11. Statistical Analyses

All of the data were expressed as the means ± SD and analyzed by analysis of variance and a Student-Newman-Keuls test using the SPSS13.0 software (SPSS Inc., Chicago, IL, USA). *p*-Values of less than 0.05 were considered to be statistically significant.

## 5. Conclusions

In this study, we discovered a new and beneficial role for PEDF, which prevented hypoxia and ischemia-induced endothelial barrier dysfunction and revealed the molecular basis for PEDF-mediated protection of hypoxic RAECs. We first reported that PEDF inhibits vascular permeability in RAECs via PPARγ under the hypoxic condition and found that PEDF protected cardiomyocytes against ischemia or hypoxia-induced cell apoptosis, both *in vivo* and *in vitro* via preventing the activation of caspase-3. PEDF may rely on any of its anti-leak and anti-apoptotic protective properties to reduce myocardial infarct size and to protect cardiac function during the development of ischemic injury in an AMI rat model. PEDF may be an indispensable environmental factor in stabilizing the cardiac microenvironment and enhancing performance of the ischemic heart. Our findings may provide a novel therapeutic strategy for ischemic heart disease.

A limitation of the study is that these results only indicated the biological effects of PEDF on cardiomyocyte apoptosis and endothelial cell permeability, the connection between endothelial cell permeability and cardiomyocyte apoptosis and what the role of PEDF in this connection is are uncertain. In addition, the effects of PEDF upregulating AJs need further study; the detailed molecular mechanisms are not certain here. Therefore, future investigation is needed to clarify these detailed mechanisms underlying the cardiac protection initiated by PEDF in the ischemic heart.

## Supplementary Materials

Supplementary materials can be found at http://www.mdpi.com/1422-0067/16/03/5618/s1.
