# Supplementary material for: PEDF Improves Cardiac Function in Rats with Acute Myocardial Infarction via Inhibiting Vascular Permeability and Cardiomyocyte Apoptosis
Source: Int J Mol Sci. 2015 Mar 11;16(3):5618–34. doi: 10.3390/ijms16035618 (PMC4394496; doi:10.3390/ijms16035618)
Supplement: Supplementary file 1 [file ijms-16-05618-s001.pdf]

Supplementary Information

1. Methods

1.1. Animal Model

Male rats were used in this study because estrogen-dependent signaling has been reported to play protective roles in myocardial ischemic injury [30]. The rats were anaesthetized with sodium pentobarbital (60 mg/kg, i.p.), and maintained during anesthesia by bolus injections of sodium pentobarbital (3–6 mg/kg, i.v.) as required. A neuromuscular blocking agent, rocuronium bromide (0.6 mg/kg, i.v.), was used to allow a more stable preparation. The rats were mechanically ventilated via a rodent ventilator. Effective levels of anesthesia were obtained and maintained by observing reactions to physical stimulation such as toe pinch, as well as monitoring the pattern of respiration. The left anterior descending coronary artery (LAD) was ligated with a 6–0 silk suture (Ethicon, Johnson & Johnson, Somerville, NJ, USA) at its origin. The mortality rate within 48 h following AMI surgery was 20%. The animals were maintained on a standard diet after surgery.

1.2. Co-Immunoprecipitation

Myocardial tissues were lysed as described above in 1mL of immunoprecipitation buffer (62.5 mmol/L Tris-HCl, 100 mmol/L NaCl, 1% NP-40, 0.1% Tween 20, 1 mmol/L Na_3VO_4, pH 8.0, and protease inhibitors) for 30 min on ice. Antibody against β-catenin (1 mg/mL; Anbo Biotechnology Co., Ltd., San Francisco, CA, USA) was added to the lysate and incubated with gentle rocking overnight at 4 °C. Agarose (50 µL)-coupled anti-mouse or anti-rabbit antibody (Sigma Chemical Co., St. Louis, MO, USA) was added for an additional 2 h, and then the agarose beads were sedimented by centrifugation and washed by resuspending and pelleting 3× with 1 mL of immunoprecipitation buffer. Then, a 100 µL of 2× Laemmli buffer were added to the agarose pellet and boiled for 5 min, and 10 µL was separated on 7% SDS-PAGE and directly used for Western blot analysis with anti-VE-cadherin antibody or anti-β-catenin antibody.

2. Supplementary Figures

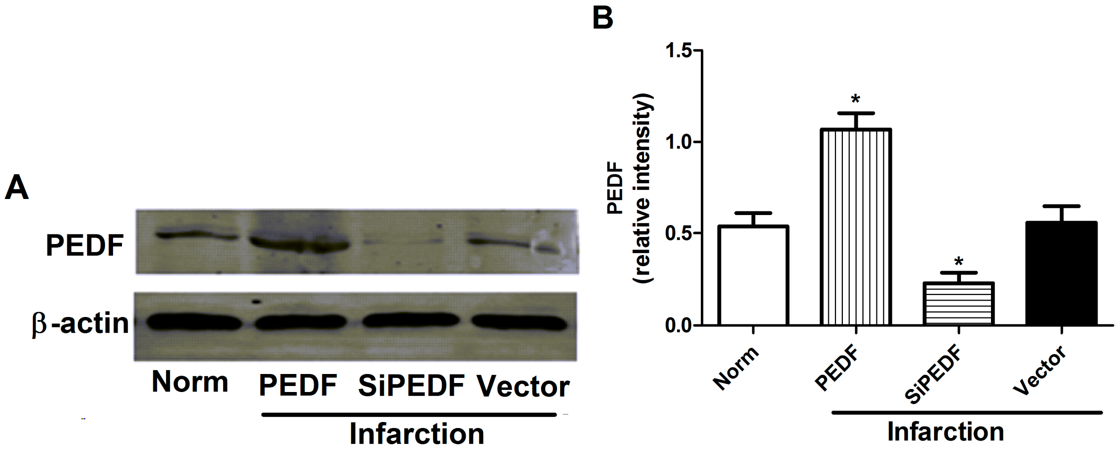

Figure S1. (**A**) western blot determination for PEDF protein expression in a rat AMI model; (**B**) Densitometry of the western blots shown in (**A**), normalized to β-actin. $n = 3$, values are means ± SD. * $p < 0.05$ *vs.* the vector control group. The results indicated that PEDF protein expression in the siPEDF group was significantly inhibited, whereas in the PEDF group, we found that an overexpression of PEDF protein was evident.

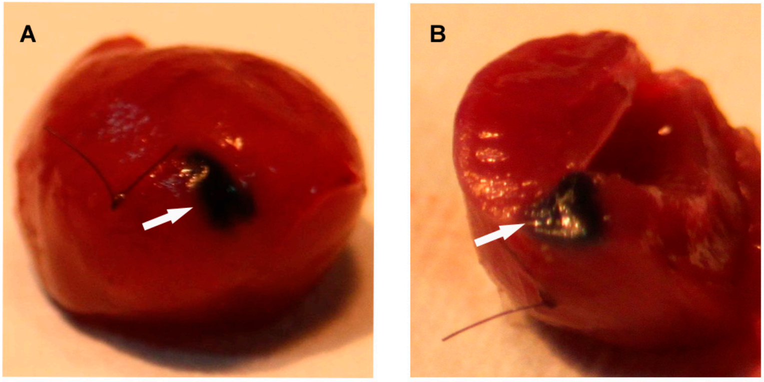

Figure S2. Methylene blue dye injection in the heart. To confirm the precise location of the injected solution, methylene blue dye was injected in order to assess the range of lentivirus vector injection in the myocardium, as seen in the actual location of the injection in histological preparation (white arrow).
